# Striking Surge in Lung Cancer Incidence in Children of Early Life

**DOI:** 10.70322/jrbtm.2026.10004

**Published:** 2026-05-28

**Authors:** Nobel Chenggong Zong, Jessica Johal, Heather Liu, Hua Cai

**Affiliations:** 1 Division of Molecular Medicine, Department of Anesthesiology and Perioperative Medicine, David Geffen School of Medicine at University of California Los Angeles, Los Angeles, CA 90095, USA; 2 Division of Cardiology, Department of Medicine, David Geffen School of Medicine at University of California Los Angeles, Los Angeles, CA 90095, USA

**Keywords:** Lung cancer, Newborns, e-Cigarette, Vaping, Pregnant women, Miscarriage, Tobacco, Radon gas

## Abstract

Lung cancer ranks first in mortality and the third in total cancer cases diagnosed in the US. The epidemiological trends may vary among different age groups, while the dynamics of risk factors evolve as well. We aim to carefully characterize trends of lung cancer among different age groups in the past two decades, by accessing the Surveillance, Epidemiology, and End Results (SEER) datasets from the National Cancer Institute (NCI), and to delineate possible root causes. The SEER datasets were obtained from NCI. Data on environmental risk factors were acquired from the Environmental Protection Agency and the United States Geological Survey. The tobacco consumption data were sourced from the Centers for Disease Control and Prevention. Trends were examined statistically with the Mann-Kendall algorithm. The incidence rate of lung cancer in the <15 age group has been rising in the past two decades, most strikingly among infants in the 0 age group (at birth to less than 1 year old). These findings were unique for lung cancer. The usage of e-Cigarettes among pregnant women increased, while the potential influence of other known risk factors was on the decline. A shrinkage of the infant population and a higher rate of pregnancy loss were observed during the same timespan. A striking rise in lung cancer incidence among infants has been identified that is opposite to the declining trend in the overall population, which might be related to increased e-Cigarette use in pregnant women. Urgent further investigation is warranted to safeguard the newborn population from being continuously affected potentially by lung cancer.

## 1. Introduction

Lung and bronchus cancer ranks first in mortality (125,070 cases estimated in 2024, 20.4% of all cancer deaths) and the third of total cancer cases diagnosed in the US [1]. Globally, lung cancer ranks first both in incidence and mortality, with an estimated 2.48 million new cases and 1.82 million deaths annually [2]. They can be broadly classified as non-small-cell lung cancer and small-cell lung cancer. Adenocarcinomas are the most common type of non-small-cell, followed by squamous-cell carcinomas [3]. Small-cell lung cancer is strongly associated with smoking and exhibits aggressive characteristics. In the latest 2023 Surveillance, Epidemiology, and End Results (SEER) report by the National Cancer Institute, the incidence of lung and bronchus cancer was in decline from 2012 to 2021 (48.8 to 39.0 cases per 100,000 population), a 20.1% reduction in ten years.

Age is a major factor relevant to the incidence rate of lung cancer. In a nutshell, the accumulated exposures towards detrimental stimuli (e.g., cigarette consumption, Radon gas inhalation) increase with age, and so does the overall vulnerability for carcinogenesis. Meanwhile, environmental and behavioral risk factors associated with lung cancer have been studied extensively. Besides smoking of combustible cigarettes, other known influences include second-hand smoke, air pollution, diesel exhaust, asbestos inhalation, radon gas exposure, and radiation [4]. In rates of lung and bronchus cancer diagnoses, it is of no surprise that the age group of senior citizens (75+) leads new retirees (65–74), then middle-aged adults (40–64), then young adults (15–39), and finally children (<15) in the US [1]. From a gender perspective, the incidence was markedly higher among males in the 75+, 65–74, and 40–64 age groups consistently from 2000 to 2021 [1]. On the other hand, females led males in the 15–39 age group during the prime childbearing years. In the <15 age group, statistics showed a higher incidence rate among males between 2000 and 2013, and since 2013 the incidence rate has been higher among females [1].

It is important to note that the incidence rate of lung and bronchus cancer in the <15 age group was quite different from all other aforementioned age groups by trending upward [1]. More strikingly, however, it is essentially unknown which subgroups of our children face the greatest liability. Identifying the most at-risk population and dissecting the root causes will help us better protect our children in the present and for the future.

## 2. Methods

### Data Source:

Data on cancer diagnoses were obtained from the Surveillance, Epidemiology, and End Results database provided by the National Cancer Institute [1]. Specifically, the dataset [Incidence—SEER Research Plus Limited-Field Data, 22 Registries November 2023 Sub. (2000–2021)] was downloaded and processed with SEER*Stat 8.4.4 software package, also available at the National Cancer Institute. Data on air pollution (ozone and carbon monoxide, 2000–2020) were downloaded from the United States Environment Protection Agency. Data on asbestos consumption (2000–2020) were obtained from the United States Geological Survey [5]. Data on smoking incidence among US adults, including pregnant women, was based on Behavioral Risk Factor Surveillance Survey datasets compiled each year by the Centers for Disease Control and Prevention [6]. Data on pregnancy loss were retrieved from the National Survey of Family Growth archive [7] curated by the Centers for Disease Control and Prevention.

### IRB approval:

We accessed de-identified datasets from public databases following the instructions of the sites, which do not require separate institutional approval for the data analyses.

### Data Processing:

With the SEER*Stat application, ‘Rate Session’ Analyses were conducted. In the ‘Data’ tab, the aforementioned dataset was selected, and ‘Age Variable’ was set as ‘Age recode with single ages and 85+’. In the ‘Statistic’ tab, ‘Rates (Age-Adjusted)’ was checked in the ‘Statistic’ panel, while ‘2000 US Std Population (single ages to 84—Census P25–113)’ was selected as ‘Standard Population’ of the ‘Parameters’ panel. In the ‘Selection’ Tab, ‘Malignant Behavior’ was checked, ‘Age at Diagnosis’ and ‘Year of Diagnosis’ selected, and ‘Site and Morphology. Site recode ICD-0–3/WHO 2008’ set to ‘Lung and Bronchus’. In the ‘Table’ tab, ‘Year of Diagnosis’ was selected as ‘Row’, while ‘Age recode with single ages and 85+’ as ‘Column’. Accordingly, age-specific data on positive diagnoses of lung and bronchus cancer were extracted. To delineate the origin and pathological features of each case, ‘Primary Site’ and ‘Laterity’ were selected in parallel analyses. The output was a table with ‘Rate’ and ‘Count’ of diagnoses each year in the form of a spreadsheet. ‘Rate’ was displayed as the number of incidents per 100,000 people.

### Statistical Analysis:

To gauge the significance of data trends over the years during the study period, Mann-Kendall test [8] was conducted with RStudio version 4.3.2 using the Kendall package (version 2.2.1). The output would have a *p* value indicative of statistical significance on a change in trend, and a tau (*τ*) value (ranging from −1 to +1) indicative of the direction and magnitude of this trend. To fit the data curve to a mathematic algorithm, linear, exponential, and polynomial models were considered. The coefficients of the models were determined using the least squares regression. Polynomial models were limited to two degrees to avoid overfitting.

## 3. Results

According to SEER*Explorer [1], it seems clear that the incidence rate of the age group 75+ is higher than that of the age group 65–74, then the age group 40–64, then the age group 15–39, then the age group <15, each year from 2000 to 2021. From the perspective of data trends, the <15 group showed a statistically significant increase during this period, as determined by the Joinpoint Trend Analysis Software (version 4.9.1.0) used by the National Cancer Institute [9].

### 3.1. Lung Cancer Incidence Increased Among Infants

**Figure 1.**
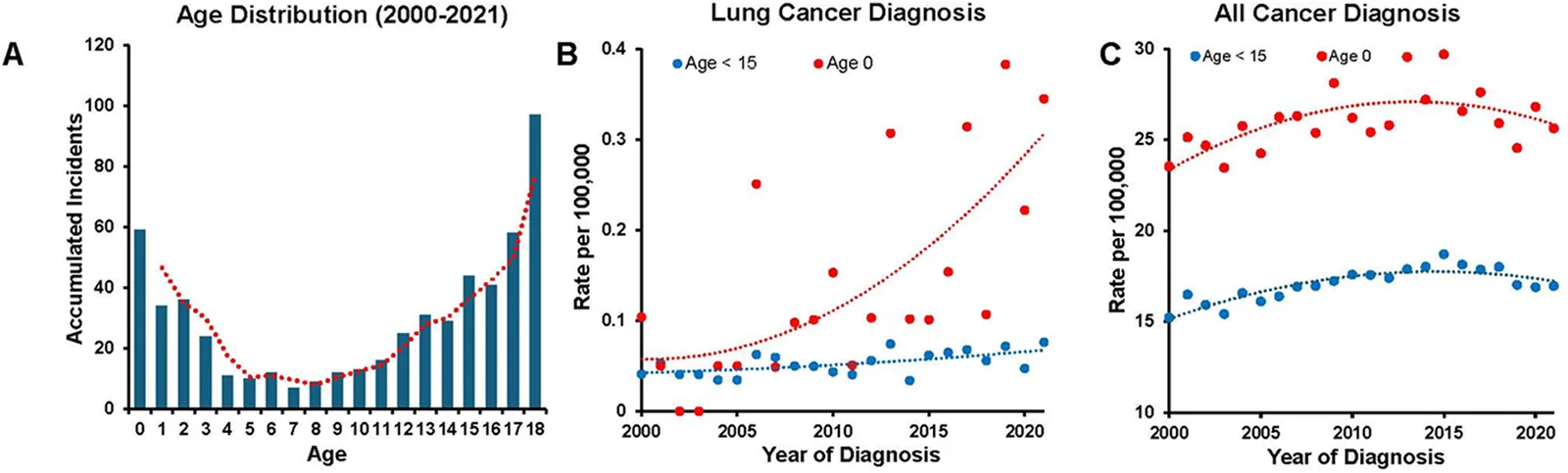
Epidemiology of lung and bronchus cancer diagnoses among children from 2000 to 2021. Graphs were composed upon SEER Research Plus Limited-field Data, 22 Registries, November 2023 Submission from the National Cancer Institute (NCI) with SEER*Stat software workstation version 8.4.3. (**A**) The accumulated numbers of lung and bronchus cancer diagnoses for each age group (0–18, one year apart) between 2001 and 2021 were plotted. A peak of diagnosis was seen at the age 0 group, then going downward and bounding back from the age 8 group till the age 18 group. A moving average trend line was illustrated in red. (**B**) The incidence rates of lung cancer diagnosis at age 0 per 100,000 population were marked with red dots; while rates of diagnosis of <15 age group per 100,000 population were marked with blue dots. Polynomial trend line for each group was plotted in color consistent with corresponding dots. Lung and bronchus cancer rates fluctuate year-by-year. Trend lines were for both groups were rising over the years, which the rates of the age 0 group increased faster. (**C**) The trend lines for all cancer diagnoses for both the age 0 group and <15 group exhibited dome-shaped curve with an apex in 2015.

Observing the rising lung cancer trend in the <15 age group, we zoomed in and examined epidemiological data from the youngest population in society to systematically interrogate the fundamentals of this unique trend. Using downloaded SEER raw datasets, we analyzed all age groups <15 and included high schoolers up to 18 years old. Plotting rate against age (Figure 1A), the accumulated incidences of lung and broncus cancer diagnosis exhibited two peaks, one on the eldest end (18-year old, 97 cases) and the other on the youngest end (0-year old, aka. infants, 59 cases), while the numbers of cases for each age group in-between were lower than either of these two peaks. This high incidence in the age 0 group drew our particular interest, as this group has shorter exposures to detrimental stimuli than their peers.

We next examined the permutation of incidence rates for the age 0 group relative to other age groups during the 2000–2021 time frame. Mann-Kendall tests were conducted instead of using Joinpoint Trend Analysis Software, due to lack in compatibility of the latter when limited number of cases were available for analyses among the youngest age groups. The statistics showed a significant increase in incidence of lung and bronchus cancer among infants during the study period (*p* = 0.0003, *τ* = 0.5690, Table 1), while the overall incidence in the whole population (all ages) exhibited a significant decline over these 22 years (*p* = 0.0001, *τ* = −0.5931). Downward trends were also confirmed in the 75+ group (*p* = 0.0241, *τ* = −0.3506), 65–74 group (*p* = 0.0000, *τ* = −0.9567), 40–64 group (*p* = 0.0000, *τ* = −0.9740), and 15–39 group (*p* = 0.0000, *τ* = −0.8528). The <15 age group yielded a significant rise in incidence rates (*p* = 0.0208, *τ* = 0.3593). Age 15 (freshman at high school, *p* = 0.1811) and age 18 (senior at high school, *p* = 0.7561) groups, in particular, did not conform to a significant direction, suggesting the age group 0 was largely responsible for the collectively rising curve among the <15 population.

We used the least squares model to fit the trend line of each age group. Among linear, exponential, and polynomial (limited to two degrees) algorithms, polynomial fitting consistently generated the highest *R*^2^ values. Plotting the incidence rates of age 0 and <15 groups (per 100,000 people) in a single chart (Figure 1B), the age 0 group displayed a steeper incline curve. This was consistent with their respective *τ* value of the statistical analyses (Table 1).

### 3.2. Characterization of Lung Cancer in the Age 0 Group

This rise in lung and bronchus cancer incidences among the very young was truly unique. With the same SEER dataset, we extracted cancer cases of all etiologies for age 0 and <15 groups and plotted them in a chart (Figure 1C). A clearly distinct pattern was observed compared to that of lung and bronchus cancer (Figure 1B). An apex of case numbers was recorded for both age groups in 2015, with a lower rate before and after that. Pre-2015, an upward trend was observed for both <15 group (*p* = 0.0000, *τ* = 0.8095) and age 0 group (*p* = 0.0056, *τ* = 0.5429). Post-2015, a downward trend was observed for both <15 group (*p* = 0.0163, *τ* = −0.8095) and age 0 group (*p* = 0.1331, *τ* = −0.5238) (Table 1).

In the age 0 group, fifty-nine cases of lung and bronchus cancer were archived in the SEER database between 2000 and 2021. Among these cases, we extracted their primary site and laterality information when denoted. The primary site of thirty cases with labeled of upper lobes of the lung (fifteen in the right, fourteen in the left and one bilateral), four cases of the middle lobe (exclusive in the right), twenty-two cases of lower lobes (even eleven on each side), six cases of lung NOS (not otherwise specified), thirty-six cases of lymph nodes NOS, one case of trachea and zero case of main bronchus (Table 2). Taking the perspective of gender-related annotation, there are noticeable differences. Categorized by primary sites, pathological evidence in the upper lobes of the lung was found in nineteen males (ten right, nine left) and eleven females (five right, five left, one bilateral); lung NOS, six males and zero females; lymph node NOS, twenty-four males and twelve females. Meanwhile, a comparable number of cases were found in the lower lobes of the lung (male: five right, four left; female: six right, seven left) and middle lobe (two cases for either gender).

### 3.3. The Dynamics of Traditional Risk Factors Showing a Downward Trend

**Figure 2.**
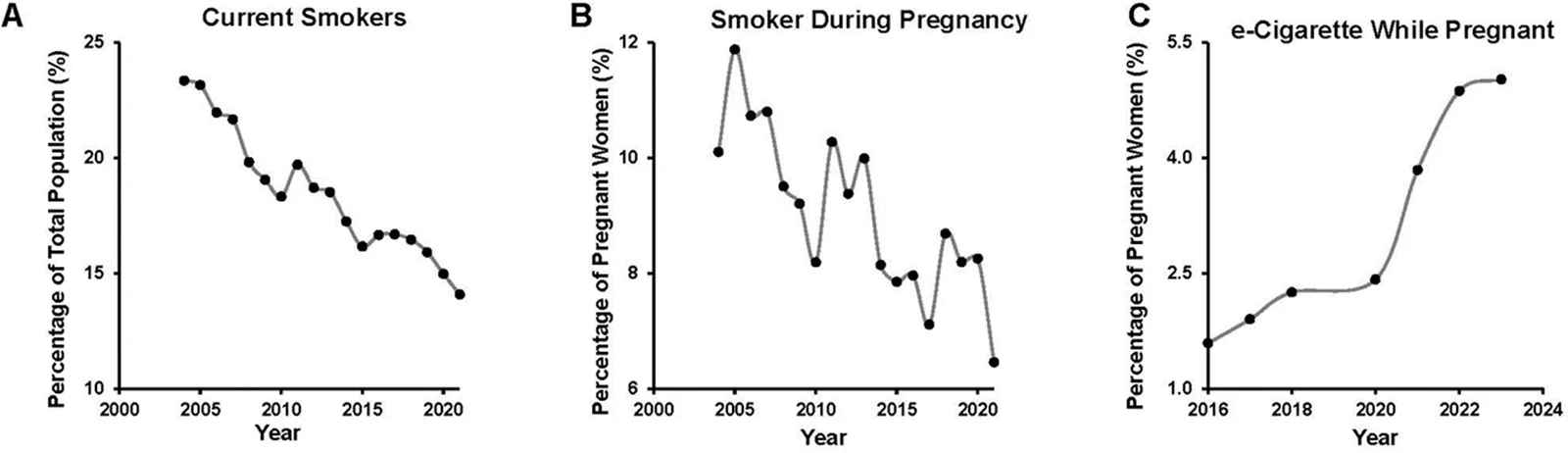
Evolvement of behavior risk factors associated with lung cancer. Charts were plotted upon BRFSS survey datasets released by CDC. (**A**) The proportions of current smokers in the whole US population were trending down from 2004 to 2021. (**B**) The proportions of women smoking during pregnancy showed higher degree of fluctuation, yet a significant decline was apparent from 2004 to 2021. (**C**) The proportion of women using e-Cigarette during pregnancy increased from 2016 to 2023.

**Figure 3.**
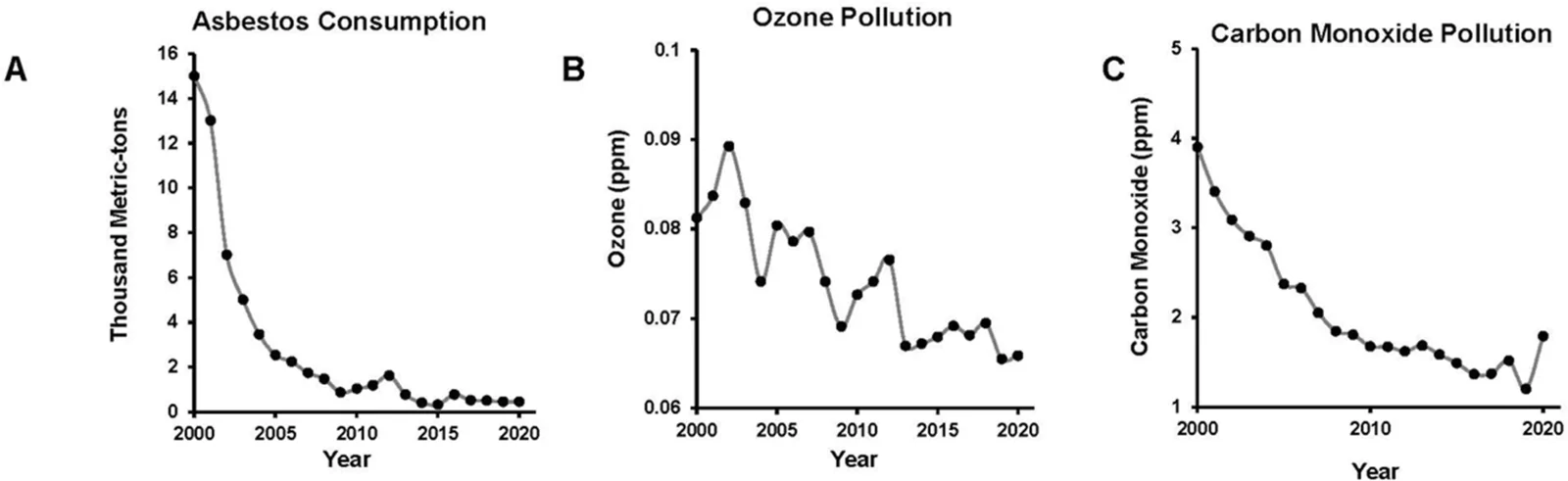
Air quality/pollution indicators associated with lung cancer risk. (**A**) The yearly total consumption of asbestos (thousands metric tons) in the US showed a remarkable decrease from 2000 to 2021 according to USGS archived datasets. (**B**) EPA released datasets on ozone levels were plotted and a gradual decline in concentration (ppm) was observed from 2000 to 2021. (**C**) EPA released datasets on carbon monoxide levels were plotted and a consistent decline in concentration (ppm) was observed from 2000 to 2021.

We examined the trends of risk factors evolving during the study period that might have impacted disease incidence. On the behavior side, the percentage of the smoking population has been drifting lower from 2004 to 2021 (*p* = 0.0000, *τ* = −0.8954, Figure 2A), based on analysis of the Behavioral Risk Factor Surveillance System (BRFSS) data collected by CDC. Importantly, this series of annual surveys also revealed that the proportion of women smoking during pregnancy was consistently lower than that of the general population and trending down (*p* = 0.0007, *τ* = −0.5948, Figure 2B). Since 2016, BRFSS questionnaires have included the usage of e-Cigarettes. From 2016 to 2023, the rate of e-Cigarette usage during pregnancy was in fact on the rise (*p* = 0.0027, *τ* = 1.0000, Figure 2C). On the environmental side, the amount of asbestos exposure was in a sharp decline between 2000 and 2020 by 97% based on data obtained from USGS (*p* = 0.0000, *τ* = −0.8353, Figure 3A). As to air quality, data from EPA indicated both ozone pollution (*p* = 0.0000, *τ* = −0.6667, Figure 3B) and carbon monoxide pollution (*p* = 0.0000, *τ* = −0.8381, Figure 3C) were in the decrease from 2000 to 2021.

**Figure 4.**
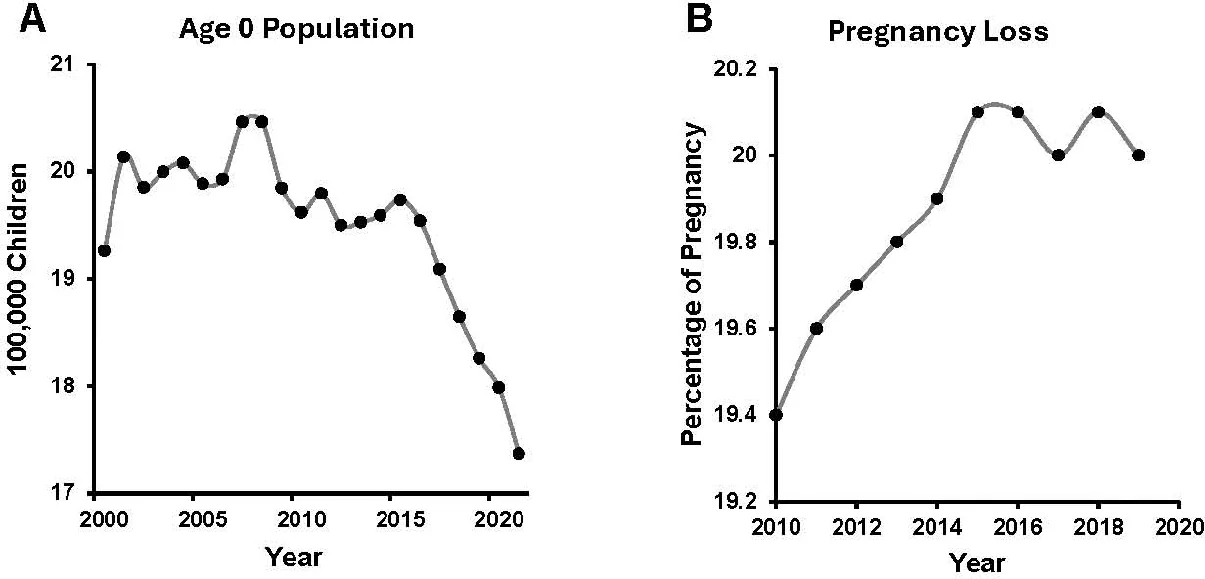
The size of age 0 population is shrinking while the proportion of failed pregnancy is on the rise. (**A**) According to SEER Research Plus Limited-field Data, 22 Registries November 2023 Submission, the size of age 0 population fluctuated and showed no significant trend before 2015 and did consistently dwindle after 2015. (**B**) NSFG datasets from CDC indicated the proportion of pregnancy ended up as losses were on the rise from 2010 to 2019. In NSFG, pregnancy loss included miscarriage, spontaneous abortion, stillbirth and fetal death.

Moreover, a significant decrease in the population size of the age 0 group was recorded in the SEER datasets. Plotting it out (Figure 4A), this trend was most remarkable starting 2015 (pre-2015: *p* = 0.1377; post-2015: *p* = 0.0027, *τ* = −1.0000). With NSFG datasets available from CDC, we investigated the rate of pregnancy losses between 2010 and 2019. The levels of losses were higher post-2015 than before (Figure 4B). The overall trend was on the rise (*p* = 0.0062, *τ* = 0.7217).

## 4. Discussion

The incidence rate of lung and bronchus cancer among infants is comprehensively analyzed in the present study, revealing a striking rise in the age 0 group (at birth to less than 1 year old), which is previously unnoticed, perhaps consequent to a seemingly overt declining incidence of lung cancer in the whole population. Rate analyses also revealed that a positive polynomial algorithm would fit this rising curve better than a linear algorithm, indicating an accelerated pace of increase. These are entirely new findings revealing a severe rising trend of lung cancer in the newborn group. Of note, adenocarcinoma of the lung in children and adolescents 18 years of age and younger were systematically analyzed previously by accessing databases from Medline, Embase, Web of Science, and Scopus, which revealed 36 cases from 48 eligible studies up to year 2018 [10]. Detailed analyses of different age groups have not been done or were not possible. From the number of absolute cases, our data also indicate a rising trend of pediatric lung cancer, especially in the age 0 group. We wish to especially emphasize on this important finding since the focus on infants aged <1 year is entirely novel and may reveal a trend that is obscured in broader pediatric or all-age lung cancer statistics.

In all forms of cancer, risk factors include family history/genetic background, and environmental and behavioral profiles, with heightened exposure in principle correlating with higher vulnerability. From the perspective of lung and bronchus cancer, the higher incidences of diagnoses among infants appear paradoxical to this paradigm. Growing up in a comparable environment, this increase is quite specific without continuing into the age groups of toddlers, preschoolers, and teenagers. Although survival rate of lung cancer in the age 0 group remains unexplored due to lack of previous recognition, this early morbidity certainly bears life-long impacts starting at a very young age. Of note, relevant to this observation, children have been shown to develop carcinoid tumors in the lung (9 cases at 8.81 ± 5 years old) [11]. From a retrospective study conducted over 8 years on children, a total of six cases of pediatric pleuropulmonary blastom (PPB) have been reported [12].

The exact reasons for this rise require thorough investigation. Regarding environmental variables, data on encounters with asbestos, ozone, and carbon monoxide pollution show persistent improvement over the years (Figure 3), as do diesel emissions reported by the EPA [13]. Thanks to environmental protection movements and legislation, over the past several decades the public has been able to avoid involuntary harm from these pollutants. Radon gas, coined as the second leading cause of lung cancer, is a radioactive decay product of Uranium-238 from the earth. Based on over 6 million measurements across the US from 2001 to 2021, a recent report indicated that a slight decrease of Radon gas levels in known high-concentration areas [14]. On behavior factors, smoking tobacco products is widely recognized as the topmost cause of lung cancer. With optimism, the society saw the proportion of active smokers declining for decades in the whole population and among pregnant women (Figure 2). However, a new form of tobacco product exposure, e-Cigarettes, has entered the market in a major way since 2015 [15–20]. Just several years later, the potential of vaping to increase lung cancer risk has been suggested by scientists [15–21]. Yet, the consumption of these novel products are on the rise in the whole population [22], adolescents [15–20], particularly alarming among pregnant women (Figure 2C). More strikingly for pregnant adolescents (10–19 years old), e-Cigarette use surged from 0.8% to 4.1% 2016–2021, while traditional cigarette use dropped from 9.2% to 3.2% 2017–2021 [20,23]. Weighted incidence analysis showed that a higher proportion of women stopped smoking upon pregnancy compared to those who stopped vaping [24]. The result of a recent study showed vaping is much less recognized as harmful or socially unacceptable compared to smoking, which is concerning to have caused unaware exposure and health consequences [25]. This gap in perception needs to be filled, especially the proportion of women vaping during pregnancy was still rising in 2023 (Figure 2C). Of note, in future investigations, it would be worthy to examine potential compounding effects of environmental exposure on e-Cigarette use, in any case territorial variations in environmental pollution would have influencing effects on top of e-Cigarette exposure.

There is growing evidence that vaping will complicate pregnancy and offspring. In animal models, chronic exposure of mice to e-Cigarettes (54 weeks) led to a 22.5% incidence of lung adenocarcinomas [26]. Exposure of pregnant mice to e-Cigarettes would also instigate chronic effects on the lung function of offspring [27]. Diving into the molecular level, there remains much to be learned. But it has been reported that e-Cigarette exposure disrupts redox homeostasis [15–20,28], alters patterns of DNA methylation and cytokine expression [29], changes the lung transcriptome [30], interferes with embryonic lung development involving a potassium ion channel [31], and impairs Wnt signaling [32]. A 2023 report showed that use of e-Cigarettes with certain flavors (mint/menthol) during pregnancy correlates with a higher rate of fetal death [33]. The scientific and clinical communities are still at an early stage in characterizing the full impact of e-Cigarettes on lung cancer etiology and epidemiology, particularly in the context of pregnancy. It is important to note that the assortment of chemicals delivered by e-Cigarettes is quite different from that of conventional cigarettes, besides nicotine. Additionally, studies on e-Cigarettes can be more difficult due to its seemingly endless variations in formulation. Pregnant women, particularly adolescents, are yet to be adequately aware of the risks to fetuses posed by e-Cigarette use. Whether fetuses are more susceptible to injury by chemicals from e-Cigarettes remains to be thoroughly examined. The confirmed cases of lung and bronchus cases reported here are likely just the tip of an iceberg. Certain fraction of rising pregnancy losses in the US during the past few years might have significant involvement of the respiratory system. Although there is not enough evidence to suggest that pregnancy loss is at least in part attributed to e-Cigarette use during pregnancy, educating pregnant women about the risks associated with vaping and reducing consumption will have a positive impact on minimizing pregnancy losses and restoring the infant population. Whereas increased e-Cigarette use during pregnancy might not be the sole reason for rising lung and bronchus cancer diagnoses in the age 0 group of infants, pregnancy losses, and shrinking size of the youngest age group, the correlation in trends and time is clear. We should be alert and recognize its potential impacts.

On e-Cigarettes and for mothers-to-be, we are and should be more vigilant this time around. According to a report from CDC 2019 Lung Injury Response Group [34], differences in nicotine concentration, flavorings infusion, formulation on other additives (e.g., propellants, solvents, and oils), as well as, the power setting of the heating element, the material used to construct the mouthpiece and atomizer, the durability of battery to hold its integrity, and chemicals used in cleaning all affect the spectrum of substances enter human lungs. Nevertheless, a habit of vaping raises the chances of elevated exposure to heavy metals (e.g., chromium, manganese, and nickel) and certain carcinogenic compounds (e.g., acrolein and propylene oxide) [15–20,28]. It is important to note that we have previously shown that e-Cigarette use directly induces endothelial dysfunction in young adults [19].

Lung cancer remains one of the top preventable diseases. Much attention has been given to smoking seniors, and we are now reaping the rewards on that front. Our next generation of young people confronts a new reality of the growing popularity of vaping and an under-informed mass of mothers-to-be. There are gaps in scientific knowledge and intervention strategies on early-age lung cancer in relation to maternal vaping. Although our data do not immediately guide changes in clinical practice, we recommend decisive investments on research and education that are urgently needed to patch these gaps up to help preserve the newborns from lung cancer loss.

## Figures and Tables

**Table 1.** Trend, rate and significance of cancer diagnoses from selected age groups.

Diagnosis	Age Groups	*p* Value	τ Value	Linear (*R*^2^)	Exponential (*R*^2^)	Polynomial (*R*^2^)
Lung and bronchus cancer	All ages	0.0001	−0.5931	0.232	0.226	0.345
	75+	0.0241	−0.3506	0.416	0.396	0.762
	65–74	0.0000	−0.9567	0.972	0.957	0.978
	40–64	0.0000	−0.9740	0.97	0.967	0.971
	15–39	0.0000	−0.8528	0.857	0.89	0.926
	<15	0.0208	0.3593	0.332	0.33	0.339
	18	0.7561	n.a.	0.022	−0.074	0.111
	15	0.1811	n.a.	0.131	0.019	0.200
	0	0.0003	0.5690	0.464	0.442	0.500
All cancers	15	0.0000 (2000–2015)	0.8095	0.506	0.493	0.760
		0.0163 (2015–2021)	−0.8095			
	0	0.0056 (2000–2015)	0.5429	0.219	0.212	0.439
		0.1331 (2015–2021)	n.a.			

All analyses were conducted based on Incidence—SEER Research Plus Limited-Field Data, 22 Registries November 2023 Submission (2000–2021) from NCI. Statistical significance was calculated with Mann-Kendall algorithm with RStudio. Curve fitting applied least square models and polynomial fitting is limited to the power of 2.

**Table 2.** Gender-specific divergence in primary sites and laterality of cancer pathogenesis.

		Male		Female	
Primary Site	Laterality	Count	Rate	Count	Rate
C33.9-Trachea				1	0.005
C34.0-Main bronchus					
C34.1-Upper lobe, lung	Right—origin of primary	10	0.046	5	0.024
	Left—origin of primary	9	0.041	5	0.024
	Bilateral, single primary			1	0.005
C34.2-Middle lobe, lung	Right—origin of primary	2	0.009	2	0.01
C34.3-Lower lobe, lung	Right—origin of primary	5	0.023	6	0.029
	Left—origin of primary	4	0.018	7	0.033
C34.9-Lung, NOS	Right—origin of primary	1	0.005		
	Left—origin of primary				
	Bilateral, single primary	4	0.018		
	Paired site, but no information concerning laterality	1	0.005		
C77.9-Lymph node, NOS		24	0.11	12	0.057

Rate is presented as the number of positive cases per 100,000 population.

## Data Availability

All of the data presented in the article are available upon reasonable requests.

## References

[1] SEER*Explorer: An Interactive Website for SEER Cancer Statistics. Surveillance Research Program, National Cancer Institute. 17 April 2024. [updated: 27 June 2024; cited 17 July 2024]. (Data Source(s): SEER Incidence Data, November 2023 Submission (1975–2021), SEER 22 registries). Available online: https://seer.cancer.gov/statistics-network/explorer/ (accessed on 17 July 2024).

[2] WHO. International Agency for Research on Cancer. Global Cancer Observatory: Cancer Today. Available online: https://gco.iarc.fr/today (accessed on 19 February 2025).

[3] Thai AA, Solomon BJ, Sequist LV, Gainor JF, Heist RS. Lung cancer. Lancet 2021, 398, 535–554. DOI:10.1016/S0140-6736(21)00312-3 34273294

[4] Cancer Facts and Figures 2023. American Cancer Society. Available online: https://www.cancer.org/content/dam/cancer-org/research/cancer-facts-and-statistics/annual-cancer-facts-and-figures/2023/2023-cancer-facts-and-figures.pdf (accessed on 2 February 2025).

[5] USGS. Mineral Commodity Summaries. US Geological Survey. January 2022. Available online: https://pubs.usgs.gov/periodicals/mcs2021/mcs2021-asbestos.pdf (accessed on 2 February 2025).

[6] Rolle-Lake L, Robbins E. Behavioral Risk Factor Surveillance System; StatPearls: Treasure Island, FL, USA, 2025. 31971707

[7] Martinez GM, Daniels K. Fertility of Men and Women Aged 15–49 in the United States: National Survey of Family Growth, 2015–2019. Natl. Health Stat. Rep. 2023, 179. DOI:10.15620/cdc:122080 36692386

[8] Kendall MG, Gibbons JD. Rank Correlation Methods, 5th ed.; Arnold E, Ed.; Oxford University Press: Oxford, UK, 1990.

[9] NCI. Lung and Bronchus, Recent Trends in SEER Relative Survival Rates, 2000–2022. Available online: https://seer.cancer.gov/statfacts/html/lungb.html(accessed on 22 April 2025).

[10] Balzer BWR, Loo C, Lewis CR, Trahair TN, Anazodo AC. Adenocarcinoma of the Lung in Childhood and Adolescence: A Systematic Review. J. Thorac. Oncol. 2018, 13, 1832–1841. DOI:10.1016/j.jtho.2018.08.2020 30194036

[11] Borgia P, Cafferata B, Paratore C, Anfigeno L, Conte A, Florio A, Primary Lung Tumors in Children: Insights from a Single-Center Case Series. J. Clin. Med. 2025, 14, 2173. DOI:10.3390/jcm14072173 PMC11989418 40217628

[12] Gupta P, Chugh S, Gupta N, Gupta K, Sodhi KS, Kakkar N, Cytomorphologic and immunocytochemical characterization of pediatric pleuropulmonary blastoma with a comprehensive review of the literature. Diagn. Cytopathol. 2024, 52, 103–115. DOI:10.1002/dc.25254 37964698

[13] EPA. Highlights of the Diesel Emissions Reduction Program (2008–2018). In Diesel Emissions Reduction Act (DERA) Fifth Report to Congress; U.S. Environmental Protection Agency (EPA): Washington, DC, USA, 2022.

[14] Li L, Coull BA, Zilli Vieira CL, Koutrakis P. High-resolution national radon maps based on massive indoor measurements in the United States. Proc. Natl. Acad. Sci. USA 2025, 122, e2408084121. DOI:10.1073/pnas.2408084121 PMC11759897 39808659

[15] Cai H, Wang C. Graphical review: The redox dark side of e-cigarettes; exposure to oxidants and public health concerns. Redox Biol. 2017, 13, 402–406. DOI:10.1016/j.redox.2017.05.013 PMC5493817 28667909

[16] Cai H, Wang C. Surviving with Smog and Smoke: Precision Interventions? Chest 2017, 152, 925–929. DOI:10.1016/j.chest.2017.06.030 PMC5812760 28694198

[17] Cai H, Garcia JGN, Wang C. More to Add to E-Cigarette Regulations: Unified Approaches. Chest 2020, 157, 771–773. DOI:10.1016/j.chest.2019.11.024 32252929

[18] Zhang Y, Wang L, Mutlu GM, Cai H. More to Explore: Further Definition of Risk Factors for COPD—Differential Gender Difference, Modest Elevation in PM_2.5_, and e-Cigarette Use. Front. Physiol. 2021, 12, 669152. DOI:10.3389/fphys.2021.669152 PMC8131967 34025456

[19] Youn JY, Middlekauff HR, Reudiseuli I, Huang K, Cai H. Endothelial damage in young adult e-cigarette users. Redox Biol. 2023, 62, 102688. DOI:10.1016/j.redox.2023.102688 PMC10121999 37018969

[20] Zong NC, Zhang Y, Cai H. Keep Gimmicks of Toy E-Cigarettes Away and Juveniles on Track Towards the Tobacco Endgame. Chest 2024, 166, 1293–1295. DOI:10.1016/j.chest.2024.06.3810 39663036

[21] Bracken-Clarke D, Kapoor D, Baird AM, Buchanan PJ, Gately K, Cuffe S, Vaping and lung cancer—A review of current data and recommendations. Lung Cancer 2021, 153, 11–20. DOI:10.1016/j.lungcan.2020.12.030 33429159

[22] Ali FRM, Seidenberg AB, Crane E, Seaman E, Tynan MA, Marynak K. E-cigarette Unit Sales by Product and Flavor Type, and Top-Selling Brands, United States, 2020–2022. MMWR Morb. Mortal. Wkly. Rep. 2023, 72, 672–677. DOI:10.15585/mmwr.mm7225a1 PMC10328473 37347717

[23] Wen X, Liu L, Moe AA, Ormond IK, Shuren CC, Scott IN, Use of E-Cigarettes and Cigarettes During Late Pregnancy Among Adolescents. JAMA Netw. Open 2023, 6, e2347407. DOI:10.1001/jamanetworkopen.2023.47407 PMC10719752 38091042

[24] Liu B, Xu G, Rong S, Santillan DA, Santillan MK, Snetselaar LG, National Estimates of e-Cigarette Use Among Pregnant and Nonpregnant Women of Reproductive Age in the United States, 2014–2017. JAMA Pediatr. 2019, 173, 600–602. DOI:10.1001/jamapediatrics.2019.0658 PMC6547070 31034001

[25] East KA, Hitchman SC, McNeil A, Thrasher JF, Hammond D. Social norms towards smoking and vaping and associations with product use among youth in England, Canada, and the US. Drug Alcohol Depend. 2019, 205, 107635. DOI:10.1016/j.drugalcdep.2019.107635 PMC6905149 31765990

[26] Tang MS, Wu XR, Lee HW, Xia Y, Deng FM, Moreira AL, Electronic-cigarette smoke induces lung adenocarcinoma and bladder urothelial hyperplasia in mice. Proc. Natl. Acad. Sci. USA 2019, 116, 21727–21731. DOI:10.1073/pnas.1911321116 PMC6815158 31591243

[27] Aslaner DM, Alghothani O, Saldana TA, Ezell KG, Yallourakis MD, MacKenzie DM, E-cigarette vapor exposure in utero causes long-term pulmonary effects in offspring. Am. J. Physiol. Lung Cell. Mol. Physiol. 2022, 323, L676–L682. DOI:10.1152/ajplung.00233.2022 PMC9722245 36218276

[28] Liu Z, Zhang Y, Youn JY, Zhang Y, Makino A, Yuan JX, Flavored and Nicotine-Containing E-Cigarettes Induce Impaired Angiogenesis and Diabetic Wound Healing via Increased Endothelial Oxidative Stress and Reduced NO Bioavailability. Antioxidants 2022, 11, 904. DOI:10.3390/antiox11050904 PMC9137638 35624768

[29] Chen H, Li G, Chan YL, Chapman DG, Sukjamnong S, Nguyen T, Maternal E-Cigarette Exposure in Mice Alters DNA Methylation and Lung Cytokine Expression in Offspring. Am. J. Respir. Cell Mol. Biol. 2018, 58, 366–377. DOI:10.1165/rcmb.2017-0206RC 28960086

[30] Noel A, Yilmaz S, Farrow T, Schexnayder M, Eickelberg O, Jelesijevic T. Sex-Specific Alterations of the Lung Transcriptome at Birth in Mouse Offspring Prenatally Exposed to Vanilla-Flavored E-Cigarette Aerosols and Enhanced Susceptibility to Asthma. Int. J. Environ. Res. Public Health 2023, 20, 3710. DOI:10.3390/ijerph20043710 PMC9967225 36834405

[31] Ozekin YH, Saal ML, Pineda RH, Moehn K, Ordonez-Erives MA, Delgado Figueroa MF, Intrauterine exposure to nicotine through maternal vaping disrupts embryonic lung and skeletal development via the Kcnj2 potassium channel. Dev. Biol. 2023, 501, 111–123. DOI:10.1016/j.ydbio.2023.06.002 PMC10445547 37353105

[32] Noel A, Hansen S, Zaman A, Perveen Z, Pinkston R, Hossain E, In utero exposures to electronic-cigarette aerosols impair the Wnt signaling during mouse lung development. Am. J. Physiol. Lung Cell. Mol. Physiol. 2020, 318, L705–L722. DOI:10.1152/ajplung.00408.2019 32083945

[33] Lin SY, Wang L, Zhou W, Kitsantas P, Wen X, Xue H. E-cigarette use during pregnancy and its association with adverse birth outcomes in the US. Prev. Med. 2023, 166, 107375. DOI:10.1016/j.ypmed.2022.107375 PMC9888444 36481272

[34] Schier JG, Meiman JG, Layden J, Mikosz CA, VanFrank B, King BA, Severe Pulmonary Disease Associated with Electronic-Cigarette-Product Use—Interim Guidance. MMWR Morb. Mortal. Wkly. Rep. 2019, 68, 787–790. DOI:10.15585/mmwr.mm6836e2 PMC6755818 31513561

